# Viability of brown trout embryos positively linked to melanin-based but negatively to carotenoid-based colours of their fathers

**DOI:** 10.1098/rspb.2008.0072

**Published:** 2008-04-29

**Authors:** Claus Wedekind, Alain Jacob, Guillaume Evanno, Sébastien Nusslé, Rudolf Müller

**Affiliations:** 1 Department of Ecology and Evolution, Biophore, University of Lausanne1015 Lausanne, Switzerland; 2 Eawag, Swiss Federal Institute of Aquatic Science and Technology6047 Kastanienbaum, Switzerland

**Keywords:** salmonid, genetic load, good-genes sexual selection, offspring survival, heritability of colour traits

## Abstract

‘Good-genes’ models of sexual selection predict significant additive genetic variation for fitness-correlated traits within populations to be revealed by phenotypic traits. To test this prediction, we sampled brown trout (*Salmo trutta*) from their natural spawning place, analysed their carotenoid-based red and melanin-based dark skin colours and tested whether these colours can be used to predict offspring viability. We produced half-sib families by *in vitro* fertilization, reared the resulting embryos under standardized conditions, released the hatchlings into a streamlet and identified the surviving juveniles 20 months later with microsatellite markers. Embryo viability was revealed by the sires' dark pigmentation: darker males sired more viable offspring. However, the sires' red coloration correlated negatively with embryo survival. Our study demonstrates that genetic variation for fitness-correlated traits is revealed by male colour traits in our study population, but contrary to predictions from other studies, intense red colours do not signal good genes.

## 1. Introduction

Some ‘good-genes’ models of sexual selection predict that attractive males or males that are superior competitors in intrasexual selection are of high genetic quality and hence offer indirect genetic benefits to females (Neff & Pitcher 2005). Empirical tests of such models need to control for potentially confounding effects of, for example, differential female investment into embryos and juveniles (Parker 2003). Therefore, the ideal species to test for genetic effects of sexual selection are those with no parental care, external fertilization and large family size, i.e. some frogs (Welch *et al*. 1998) and some fish species (e.g. Wedekind *et al*. 2001, 2004; Rudolfsen *et al*. 2005; Pitcher & Neff 2006). However, it is still unclear whether colours can be linked to genetic quality in such species.

Animals often use colours to advertise their quality and to attract mates. Two main groups have been discussed as candidates in this context (McGraw 2005; Griffith *et al*. 2006): carotenoid-based yellow or red colours and melanin-based brown, black and grey colours. The reliability of the signals they produce is assumed to be given by the costs of colour production and/or maintenance (Grafen 1990). Various non-exclusive types of costs have been discussed (Hadfield & Owens 2006) as follows: (i) the pigments are costly to acquire, e.g. because they are rare or owing to costly physiological handling (metabolism or transport), (ii) the pigments are required by other physiological processes, e.g. for immune functioning, or (iii) the pigments are toxic or the colours are dangerous, e.g. because they enhance conspicuousness to predators or they signal a social status that may need to be defended.

In vertebrates, carotenoids that are responsible for yellow and red colours have to be obtained through the diet. They may therefore reliably signal foraging quality (Olson & Owens 1998) or foraging strategy (Bakker *et al*. 1997). Such colours have often been investigated in sexual selection studies because they are usually conspicuous and based on pigments that also have antioxidant and immunoregulatory properties (McGraw 2005; Peters 2007). Hence, by using these pigments, the signaller may reveal its superior health and vigour to potential mates (McGraw & Ardia 2003). In the three-spined stickleback (*Gasterosteus aculeatus*), for example, males use conspicuous red colours to attract females (Milinski & Bakker 1990; Bakker & Mundwiler 1994). These colours are based on at least three different carotenoids (Wedekind *et al*. 1998) that are known to be important in immune function (Krinsky 1993). Accordingly, red colours in these fish reveal good condition (Frischknecht 1993; Barber *et al*. 2000) and high resistance against parasite infection (Milinski & Bakker 1990; Barber *et al*. 2001), and females benefit from preferring red males especially when males are energy constrained during their paternal care (Candolin 2000).

In species without paternal care, i.e. where males only provide genes to their offspring, the role of carotenoid-based colours in signalling and mate choice is less clear. In Arctic charr (*Salvelinus alpinus*), for example, first observations found a link between red colours and immune function (Skarstein & Folstad 1996), but these colours do not seem to be useful indicators of health and vigour or resistance against infection: Skarstein *et al*. (2005) even found a positive correlation between red coloration and parasite intensity, and Masvaer *et al*. (2004) reported a positive link between red coloration and milt characteristics that indicate low dominance in male–male interaction at the spawning place (Rudolfsen *et al*. 2006). In guppies (*Poecilia reticulata*), another fish with no paternal care, variation in carotenoid-based colour traits can be linked to infections and reactions of the immune system (Grether *et al*. 2004; Kolluru *et al*. 2006), and females have been found to prefer males with larger amounts of carotenoids in their orange spots (Grether 2000). However, female responsiveness to male colours varies with carotenoid availability (Grether *et al*. 2005) and age (Kodric-Brown & Nicoletto 2001), and females are also reported to prefer larger males to brightly coloured ones (Houde 1997).

In contrast to carotenoid-based colours, melanin-based colours can be synthesized by the animal itself (e.g. in specialized organelles called melanosomes; Sugimoto 2002) and the synthesis is under strong genetic control (Majerus & Mundy 2003). Melanin-based colours may have therefore been less obvious candidates for reliable signals of genetic quality, but it has recently been recognized that significant relationships exist between melanin-based pigmentation and overall health and vigour (Roulin 2004*a*; McGraw 2005). In birds, melanin-based traits are frequently linked to fitness traits (reviewed by Roulin (2004*b*); recent examples include Bize *et al*. (2006), Fargallo *et al*. (2007) and Roulin (2007)). These links could be due to the antioxidant activity of melanin (McGraw 2005) or its involvement in calcium, zinc and iron metabolism and in the maintenance of body structures (Niecke *et al*. 2003; Roulin *et al*. 2006; McGraw 2007). In addition, melanin plays important roles in the developmental pathways of various functions and may therefore be an indicator of homeostasis (Badyaev & Young 2004; Roulin 2004*a*). Finally, although melanin-based colours often function as camouflage, some melanin-based signals may make the signaller more conspicuous in some environments (Smith *et al*. 2004), and only animals in good health and vigour may be able to pay the costs of being conspicuous (Kodric-Brown 1998).

The brown trout is a salmonid with external fertilization and no paternal care, and with migratory and resident forms. The resident form (*Salmo trutta* resident morph) develops brown, black and red spots on the body sides and the adipose fin. The red spots on the skin and the adipose fin contain large quantities of different carotenoids (xanthophylls and astacene esters) and much more so than any other tissue studied (Steven 1948). The migratory forms of brown trout are usually larger and therefore expected to be more dominant at the spawning place (Jacob *et al*. 2007); they have dark spots but usually no red spots when they arrive at the spawning place. We chose a resident population that has not been supplemented by hatchery fish since at least 10 years. We tested whether males differ in their genetic quality, determined as their offspring viability, and if so, whether male colour traits reveal genetic quality. We also tested for heritabilities of colour traits by comparing 2-year-old juveniles with their fathers.

## 2. Material and methods

Brown trout were collected from their natural spawning place in River Enziwigger (near Willisau, Switzerland) in November by electrofishing. Fifteen mature males were narcotized, measured for size and weight and their milt stripped for the *in vitro* fertilization of the eggs of one large female of the same population. The eggs of this one female were about equally distributed to 15 Petri dishes and 20 μl of milt were added each and activated with sand-filtered lake water (8°C), following the methods of Wedekind & Müller (2004). The resulting embryos were reared in four separate Petri dishes per sibship in 50 ml sand-filtered lake water at 4.7°C (mean number of eggs per Petri dish: 22.3±12.0 s.d.; three unfertilized eggs were earlier discarded).

**Figure 1:**
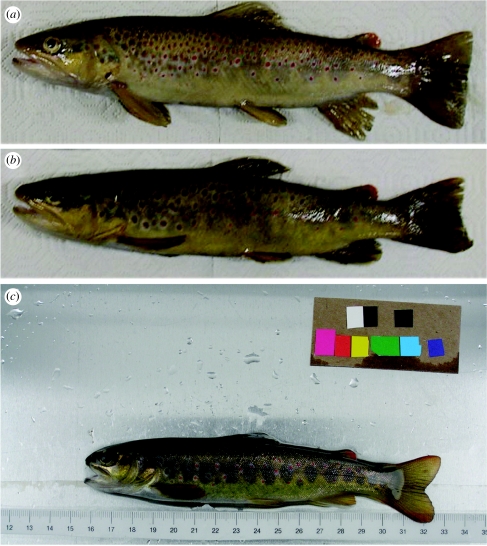
Size-standardized photos of (*a*) the male that received the highest values for red coloration but whose embryos had the lowest survival and (*b*) the male with the highest embryo survival. (*c*) Standard-distance photo of a typical juvenile that was caught back after 20 months in the wild. The reference colour patches are taken from Kodak Color Separation Guide and Grey Scale.

The males were individually marked and kept together in a large tank at approximately 8°C for another breeding experiment 14 days later (Jacob *et al*. 2007). They were then killed with a sharp blow on their head and photographed from both sides under standardized conditions with a digital camera (Nikon E995, 2048×1536 pixels; figure 1*a*,*b*). Scale samples were taken from below the adipose fin near the lateral line to estimate the age of the fish from yearly grow rings (Rifflart *et al*. 2006).

From day 60 after fertilization onwards, non-viable embryos and hatched larvae were recorded and carefully removed from the Petri dishes with a plastic spoon (in regular intervals of approx. 10 days each). Alevins were kept in darkness in running water at 7–8°C until all embryos had hatched and most alevins had nearly used up their yolk sac, i.e. until day 131 after fertilization. We then released all fish plus some additional ones from two other studies (Jacob *et al*. 2007; Evanno *et al*. submitted) into a 600 m long semi-natural, structured streamlet near Willisau that is typical for the streamlets in the region and that is confined by physical barriers. The streamlet has a width of up to 0.5 m and an average depth of approximately 10 cm. We removed all present trout by electrofishing and released our fish by carefully distributing them over the full length of the streamlet during a period when water discharge was low and not obviously affecting the larvae. We caught the fish back 20 months later again by electrofishing, measured their weight and length and took standard photos from both sides (Olympus C77OUZ, 2288×1712 pixels; figure 1*c*). DNA was extracted from fin clips, and eight microsatellite markers (Mst85, Mst543AE, BS131, T3-13, AETG1, Sssosl417, Ssa171 and Str58) were used for parental assignment following the procedure described by Jacob *et al*. (2007). Paternity was established by exclusion using the Cervus program (Marshall *et al*. 1998). All inferred parent–offspring pairs were checked for the absence of mismatching loci.

The photos of the parental and juvenile fish were used to count the number of red and brown or black spots. Further colour analyses were done on TIFF files in the open-access software ImageJ (http://rsb.info.nih.gov/ij/). We used a mode that defines hue, saturation and brightness for every pixel of the image (the ‘HSB mode’). Hue is given as an angle on a continuous circular scale from 0° to 360°, with 0° for red, 60° for yellow, 120° for green, 180° for cyan, 240° for blue and 300° for magenta. Saturation is the purity of the colour from 0% (grey) to 100% (fully saturated colour), and brightness is the relative lightness or darkness of the colour from 0% (black) to 100% (white). In ImageJ, all three values are converted to a scale from 0 to 255, and thus every pixel is defined by three values within this range. To obtain the measurements of the red coloration of our fish, we defined *a priori* a range of reddish hue values (225°–255° and 0°–20°). We then counted all the pixels within this range to determine a fish's overall redness as the total red area on the body sides and the adipose fin divided by the total area of the body sides and the adipose fin. The average modal hue and saturation of the red coloration, i.e. the most frequent hue and saturation values within the area that was perceived as red, were used as a qualitative measure of the red pigmentation. We also determined the average modal grey value on both body sides, i.e. the most frequent grey value as a measure of overall dark pigmentation. Our methods did not, however, allow us to measure colours in the ultraviolet spectrum. See Stevens *et al*. (2007) for a discussion of colour measurements from digital photos. Figure 1 indicates the range of observed male phenotypes. The analogous measures were taken from the standard photos of the 2-year-old offspring.

Statistical analyses were done in JMP v. 6.0 (www.jmp.com) or the open-access software R (www.r-project.org; see also Bates 2005). Pearson's correlation coefficients *r* are used when graphical inspection of the data suggested that the model assumptions of this statistics were not notably violated, otherwise Spearman's rank-order correlation coefficients *r*_s_ are used. Mixed-effects logistic models were used to test for sire effects on offspring viability. To avoid potentially confounding Petri-dish effects, we excluded all Petri dishes that could be considered as outliers, i.e. they were linked to embryo mortalities that were 1.5 times larger than the interquartile of the respective half-sib family (4 out of 60 Petri dishes). The sire effect was then assessed in two tests: first, a log-likelihood ratio test between a reference model (including both Petri dish and sire effect) and a reduced model (with only one of the two parameters); second, the generation of a log-likelihood distribution of the model under the null hypothesis by random permutation (*n*=1000) and a rank comparison of the observed likelihood with this distribution.

## 3. Results

Fertilization success was close to 100% for all males (only three out of in total 1454 eggs did not seem to contain an embryo). Embryos were clearly visible on day 60 after fertilization, with no apparent mortality until then. Total embryo survival until hatching was 86.9% (median 90%, range 20–100% per Petri dish). Males differed in the viability of their embryos (permutation test, male effect on embryo mortality: *p*=0.002; table 1).

**Figure 2:**
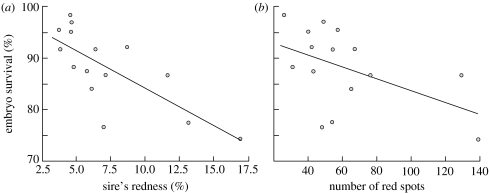
Male red colour characteristics versus embryo survival. Total embryo survival until hatching versus (*a*) overall redness, calculated as the relative red area on the body sides and the adipose fin (*r*_s_=−0.76, *p*=0.0009), and (*b*) total number of red spots on both the body sides (*r*_s_=−0.52, *p*=0.048). The lines give the regressions.

**Figure 3:**
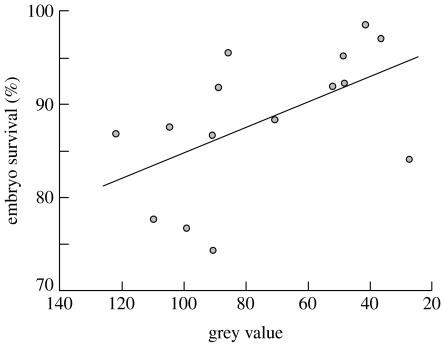
Modal grey value (most frequently occurring grey value, average of two body sides) versus embryo survival (regression line; *r*_s_=−0.60, *p*=0.018). Low modal grey values indicate dark overall pigmentation.

All red coloration measures seemed to be independent of male size and age (the sires' overall redness, the number of red spots or red hue and saturation versus age or size measures: −0.39<*r*_s_<0.11, *n*=15, always *p*>0.05). Embryo viability was negatively correlated with the sires' overall redness and the number of red spots (figure 2). Embryo survival increased with increasing dark pigmentation of their fathers as revealed by low modal grey values (figure 3). Increasing dark pigmentation was also linked to male age (*r*_s_=−0.53, *p*=0.04) and male weight (*r*_s_=−0.51, *p*=0.05), but embryo survival could not significantly be predicted by male age or size (0.002<*r*_s_<0.18, always *p*>0.05).

Out of 1261 hatchlings that were released into the wild, 10 juveniles (0.8%) could be caught back 20 months later. They were sired by eight males that did not differ significantly from the other seven males in age or in any of the colour or size measurements (Kruskal–Wallis, always *p*>0.05). There was also no significant correlation between juveniles' size or condition factor and the sires' age, size or colour traits (Spearman's rank-order correlations, always *p*>0.05). The low recapture rate remains unexplained but could be linked to an extraordinary high water that occurred few months before recapture and that caused strong currents at the study site.

**Figure 4:**
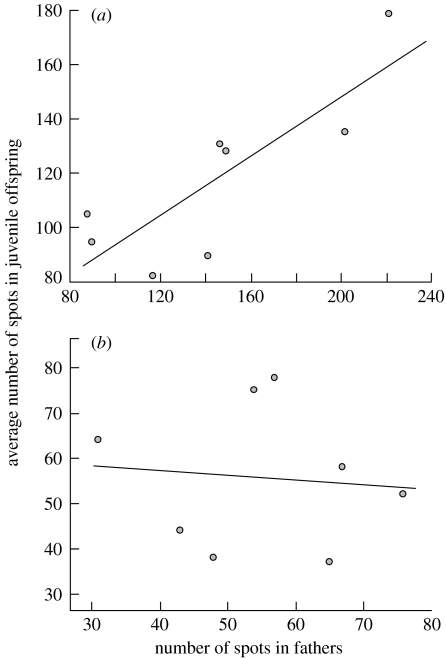
Regression of sires' spottiness versus average spottiness of their juvenile offspring. Total number of (*a*) spots on both body sides (*r*=0.83, *p*=0.01) and (*b*) red spots only (*r*=−0.09, *p*>0.05).

Spottiness, i.e. the total number of black, brown and red spots on both body sides, appeared to be heritable (figure 4*a*), while no evidence for a heritability of other pigmentation traits was found (correlation between sire and offspring, overall dark pigmentation: *r*=−0.28, *p*>0.05; overall redness: *r*=−0.23, *n*=8, *p*>0.05; red spots, figure 4*b*). We found no variation in the hue value of the red colour patches among the juveniles that could be recaptured.

## 4. Discussion

In fishes, nuptial coloration can function both in inter- and intrasexual selection (Kodric-Brown 1998). In the case of the brown trout, the relative importance of agonistic interactions between males and courtship to females is still unclear. It is even unclear whether there is any female mate choice in the brown trout (Petersson *et al*. 1999; Jacob *et al*. 2007). However, generalizations about a species' breeding system are difficult to draw because fishes often respond opportunistically to temporal or spatial variation in sex ratio, population density, habitat structure and other ecological factors (Kodric-Brown 1998). Our results suggest that if females choose and base their mate choice on colours, they should prefer darker males in order to increase the viability of their embryos. Contrary to what could be found in many other species (see §1 and below), they should not prefer red males. In fact, they should even avoid mating with bright and red males owing to the low viability of the embryos these males sire. However, we could not detect any positive or negative links between sire colour characteristics and the viability of their juvenile offspring. This could be due to either low statistical power or the different nature of embryo and juvenile mortality (Heath *et al*. 1999).

In salmonids, females are usually restricted in their choice of mates. They often defend spawning territories where they spawn their eggs in several batches, and males compete for access to these females (Fleming & Reynolds 2004; Quinn 2005). Large males are more successful in such interactions, and females seem to prefer larger males as they deposit more eggs with larger males (Foote 1989), and they are more aggressive towards small males than towards large males (Allen *et al*. 2007) or may delay spawning when courted by non-preferred males (Esteve 2005). Some *Oncorhynchus* species, especially the anadromous and non-anadromous forms of *Oncorhynchus nerka* (sockeye salmon and kokanee, respectively) or *Oncorhynchus mykiss* (steelhead and rainbow trout, respectively), develop intense red colours during the spawning season. These colours are carotenoid based (Bjerkeng *et al*. 1992; Craig *et al*. 2005), and, as in the three-spined stickleback (see §1), the red breeding colour of *O. nerka* has been found to positively influence mate choice (Craig & Foote 2001; Foote *et al*. 2004).

Animals often announce their aggressive stage and their fighting ability in colours and other characteristics (Huntingford & Turner 1987; Roulin 2004*b*), and fighting ability could be correlated with overall health and vigour and hence with good genes. However, it is not yet clear whether darker skin colours signal dominance or subordination in brown trout. Among juvenile fishes, social subordination has been linked to darker skin colours in rainbow trout (*O. mykiss*; Abbott *et al*. 1985), Atlantic salmon (*Salmo salar*; O'Connor *et al*. 1999) and Arctic charr (*S. alpinus*; Hoglund *et al*. 2000). Among spawners, however, the situation could be very different (Fleming & Reynolds 2004; Esteve 2005). A change to dark colours that may signal dominance or attractiveness during the spawning season is often seen in freshwater fishes (Kodric-Brown 1998).

There are different types of fish chromatophores, including melanophores that contain melanized organelles and that produce dark colours on the skin (Fujii 1993; Sugimoto 2002). Some chromatophores show very motile responses, for example, in response to their surroundings (some fishes are dark on a black background and pale on a white background; Sugimoto 2002). The variation in colours that we observed is, however, unlikely to reflect responses of the fish to their light environment. We controlled for such environmental variation by keeping the males for 14 days in one large unstructured tank before they were photographed.

Carotenoid-based colours can have a genetic basis (Craig *et al*. 2005), but the within-population variation is often due to diet and factors that influence carotenoid uptake and storage (Craig *et al*. 2005; Hadfield & Owens 2006). Darker skin patterns are usually due to higher melanin content (Hoglund *et al*. 2000; Pavlidis *et al*. 2006). Compared with carotenoid-based colours, melanin-based colours seem to have a stronger genetic basis (Hudon 1994; Hadfield & Owens 2006), but environmental influences are nevertheless possible (Hoglund *et al*. 2000; Fargallo *et al*. 2007). We found a significant father–offspring correlation in the total number of spots, i.e. this aspect of the skin coloration appears to be heritable. An analogous heritability has been observed in rainbow trout (Kause *et al*. 2003). We found no significant evidence for any heritability of red colour traits, but since our heritability analyses are based on a rather small sample size, such non-significant findings cannot be taken as evidence for a lack of heritability.

In a recent study on brown trout, Forsberg *et al*. (2007, p. 1863) found ‘… no apparent reproductive skew that would have indicated a strong ‘good genes’ effect’. However, they also found that their most successful males had a higher ‘microsatellite score’, i.e. these males were more different in their average genetics from the rest of the sample than males with lower reproductive success. It remains unclear whether this microsatellite score is linked to variation in genetic load or to the origin of the breeders (the authors used a mixture of hatchery- and wild-born males). In the former case, within-population variation in mating success may be caused by variation in genetic load. Our findings would then suggest that the genetic load is revealed by melanin-based colour traits. However, contrary to other species, a mate preference for red colours would not promote indirect genetic benefits in the case of the brown trout.

## Figures and Tables

**Table 1:** Sire effects on embryo survival. (Three mixed-effects logistic models to test whether sire effects explain a significant part of the variance in embryo survival (a binary response variable; egg number *n*=1290). Model parameters are random effects. The goodness of fit is given by the logarithm of the approximated likelihood (log *L*) and the Akaike information criterion (AIC). A measure to compare the quality of fit between two models is the difference in AICs between two models. Likelihood ratio tests between the reference model and the other models are used to test which parameter significantly improves the goodness of fit.)

					likelihood ratio test against the reference model		
					
model	effect tested	model parameters	log *L*	AIC	*Χ*^2^	d.f.	*p*
reference model		Petri dish, sire	−383.79	773.58			
environmental model	sire	Petri dish	−387.15	778.31	6.73	1	0.0095
sire model	Petri dish	sire	−383.79	771.58	<0.01	1	1
